# Application of selection Index for enhancing resistance to *Cryptocarya irritans* and *Vibrio alginolyticus* in large yellow croaker

**DOI:** 10.3389/fvets.2025.1524914

**Published:** 2025-01-17

**Authors:** Jiaying Wang, Ji Zhao, Bo Liu, Hongshu Chi, Pengxing Jiang, Junzu Xiao, Zhou Jiang, Lingwei Miao, Qian He, Junjia Zeng, Qiaozhen Ke, Tao Zhou, Peng Xu

**Affiliations:** ^1^State Key Laboratory of Mariculture Breeding, College of Ocean and Earth Sciences, Xiamen University, Xiamen, China; ^2^Fujian Key Laboratory of Genetics and Breeding of Marine Organisms, College of Ocean and Earth Sciences, Xiamen University, Xiamen, China; ^3^Key Laboratory of Cultivation and High-Value Utilization of Marine Organisms, Fisheries Research Institute of Fujian, Xiamen, China; ^4^Biotechnology Institute, Fujian Academy of Agricultural Sciences, Fuzhou, Fujian, China

**Keywords:** selection index, disease resistance trait, genomic selection, large yellow croaker, aquaculture

## Abstract

Despite the high cost of genotyping and the limited economic return per individual that restricts the use of genome selection technology in aquatic species breeding, integrating multiple traits in a selection index can reduce costs and present a promising direction for fish breeding. The aim of this study was to evaluate the feasibility and performance of breeding for dual resistance traits against *C. irritans* and *V. alginolyticus* in large yellow croaker, using a selection index approach. A candidate stock population from the *C. irritans* resistance strain (GS3) of the F2 generation (*n* = 560) was genotyped using a 55 K SNP array. A total of 50 broodstock with the highest genomic estimated breeding value (GEBV) ranking for the *C. irritans* resistance trait were selected to produce the F3 generation, while 50 broodstock with the highest selection index were selected to breed the double trait strain (GS9). In parallel, two populations without selective breeding population (C1and C2) were used as the control group to produce the offspring. The genetic improvement effect of all the breeding lines (GS3, GS9, C1, and C2) was assessed through artificial challenge experiments with *C. irritans* and *V. alginolyticus*, respectively. The GWAS result indicated that resistance to *V. alginolyticus* was a polygenic trait and the heritability of resistance to *V. alginolyticus* was estimated to be approximately 0.1. The survival rates of GS9 and GS3 were 43.74 and 86.81%, respectively, after 120 h of challenge with *C. irritans* infection, whereas those of C1 and C2 remained at 0%. Furthermore, the survival rates of GS9 and GS3 were 27.03 and 15.48%, respectively, after 48 h of *V. alginolyticus* infection, while the survival rates of C1 and C2 were 12.67 and 14.17%, respectively. These results indicate that genetic improvement of multiple traits in large yellow croaker using genomic selection technology is indeed feasible. Moreover, this study provides a valuable reference for advancing multi-trait breeding applications in aquatic species.

## 1 Background

The selection index has been widely used in animal breeding since it was introduced by Hazel (1). Compared to single-trait selection, the selection index allows each trait to be weighted according to its economic value and then allows simultaneous genetic improvement of each trait to maximize the genetic effect (1). According to national conditions, a total of 13 vital traits such as milk yield, protein yield, fertility, yield and health are included in the comprehensive index for genetic improvement of dairy cattle varieties in many countries (2). Over several decades, the average milk yield has nearly doubled from 6,619 to 12,662 kg in US Holstein dairy cattle breeding, and genetic gains for milk, fat, and protein yields were 109, 6.0 and 4.1 kg per year after the implementation of genomic selection (3). Furthermore, an analysis based on the genetic progress made by dairy cattle on the selection index over the last decade to predict genetic progress over the next 50 years showed that milk yield, fat and protein could also increase by 34, 53, and 41%, respectively (2). In aquaculture, O’Flynn et al. conducted two generations of genetic improvement on Atlantic salmon, focusing on growth, seedling survival rate, and harvest weight using the selection index. The results showed an average weight gain of 0.88 kg, an increase in body length by an average of 4.48 cm, and a 9% increase in survival rate (4). Similarly, Friars et al. also demonstrated that higher genetic gains can be achieved on Atlantic salmon based on selection index compared to mass selection (5). In the blue tilapia (*Oreochromis aureus*) selective breeding program, the results indicate that after four successive generations of index selection, the selection response for growth traits in each generation reached 14%. Additionally, the survival rate of tilapia in soil pond and cold-water tolerance experiments increased by 8.4 and 1.2%, respectively (6).

As the most productive marine fish in China, the large yellow croaker (*Larimichthys crocea*) achieved a production level of 280,997 tonnes in 2024 (7). Despite this success, persistent diseases are still the primary obstacle in healthy culture of the large yellow croaker. Among these, parasitic diseases like *Cryptocarya irritans* (*C. irritans*) have significant implications on the survival rate of large yellow croaker. Currently, the application of genomic selection for resistance against *C. irritans* in large yellow croaker has shown promising results. The infection challenge experiment results indicated that the survival rate of selective lines has been significantly enhanced compared to the control group (8, 9). However, the single trait genetic improvement of resistance against *C. irritans* in large yellow croaker does not sufficiently address the complex environmental challenges in the culture region, such as bacterial diseases.

*Vibrio alginolyticus* (*V. alginolyticus*), a member of the Vibrionaceae family and the genus Vibrio, is classified as a halophilic thermotolerant, facultatively anaerobic vibrio (10). Infections of *V. alginolyticus* in large yellow croakers and the stimulation of *C. irritans* parasitism often occur concurrently during the summer. The global distribution of *V. alginolyticus* includes seawater and estuaries, with marine vibrio being its dominant species (11).

A 2013 study utilized data on the ulcerative condition of large yellow croaker from sea nets in Xiangshan County, Zhejiang Province, to perform a diagnostic tracing analysis of pathogenous vibrios. The findings revealed that the infection rate of *V. alginolyticus* was significantly higher than that of *V. harveyi* and *V. parahaemolyticus* (12). *V. alginolyticus* infects fish through attachment to compromised fish skin and oral mucosa. Infected tissues often exhibit symptoms such as congestion, inflammation, and skin erosion. Currently, there are reports of vibrio-associated diseases in the fish species such as *Larimichthys crocea* (13), *Epinephelus coioides* (14) and *Litopenaeus Vannamei* (15). Another study analyzing 116 randomly sampled seafood products from the aquatic product market in Beijing found a positive detection rate of *V. alginolyticus* at a staggering 82% (16). At present, research on controlling the disease caused by *V. alginolyticus* has led to the development of inactivated vaccines for key farmed species such as whiteleg shrimp, halibut, and pompano. However, the industry’s wide variety of cultivated species makes it challenging and costly to administer and inject thesevaccines. Therefore, starting with genetic breeding based on the selection index and integrating resistance to *V. alginolyticus* and *C. irritans* into genetic improvement target traits presents another effective approach to mitigate losses in large yellow croaker.

Prior to the establishment of a breeding line with resistant toward both *C. irritans* and *V. alginolyticus*, it is essential to devise a challenge standard for *V. alginolyticus*, assess the genetic correlation between those two disease traits and establish a reference population. The objective of this study was to explore the feasibility and practical improvement effect of using a selection index to enhance the resistant to *C. irritans* and *V. alginolyticus* traits in large yellow croaker.

## 2 Methods

### 2.1 Ethics statement

This study was approved by the Animal Care and Use committee at College of Ocean and Earth Sciences, Xiamen University. The methods were carried out in accordance with approved guidelines.

### 2.2 Technical route of breeding application

**Figure 1:**
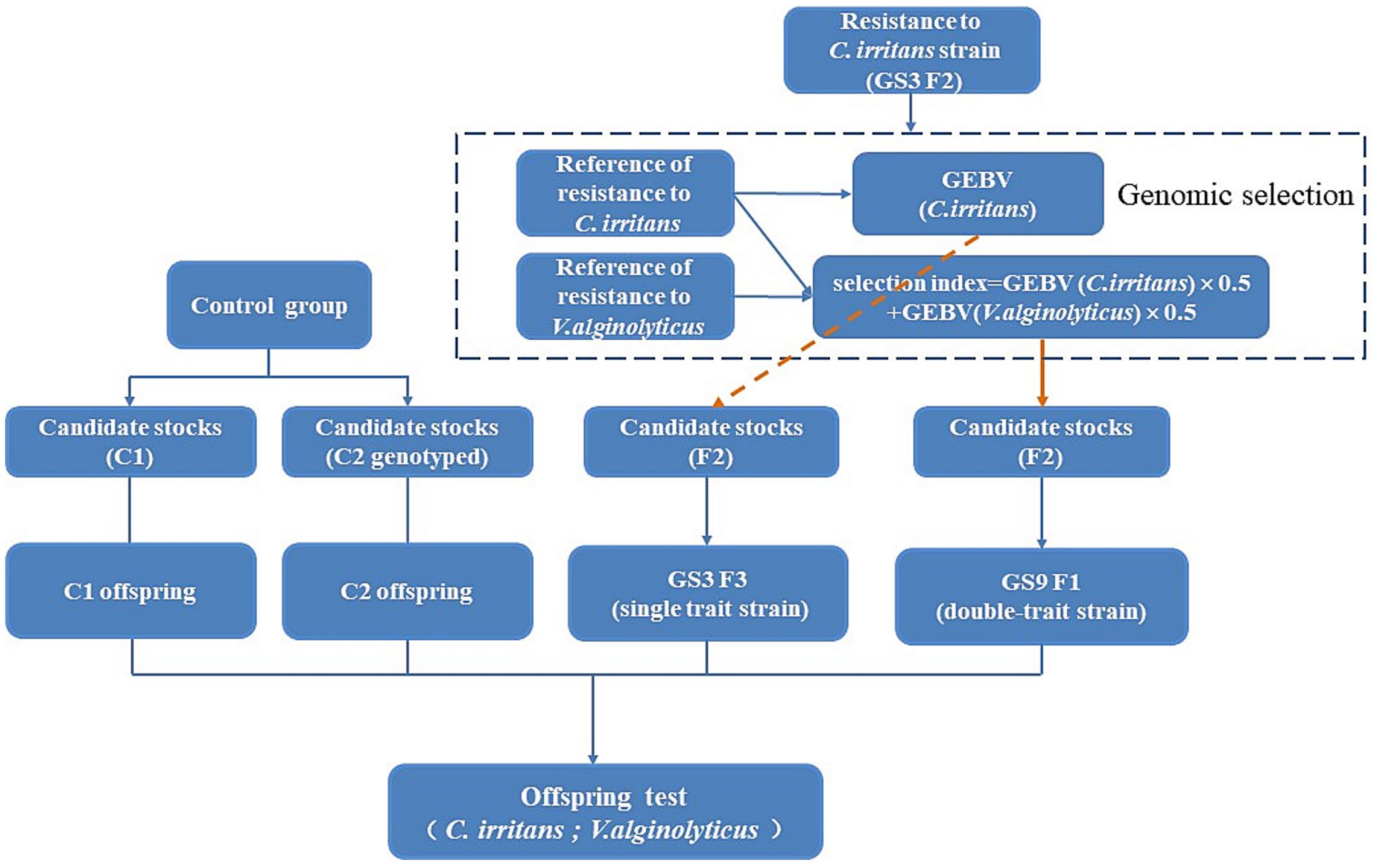
The technical roadmap of genomic selection of double trait in large yellow croaker.

The candidate stock population (*n* = 560) for genetic improvement of the dual resistance traits based on the selection index originated from the F2 generation of the breeding line designated as GS3 of the large yellow croaker, which was previously subjected to selective breeding for resistance to *C. irritans* in F1 generation (GS3) in the laboratory. The reference population (*n* = 474) for resistance to *C. irritans* in large yellow croaker was constructed based on the F1 population (GS3) in the laboratory earlier. This close genetic relationship between the reference and candidate stock populations enabled a higher accuracy in calculating the GEBV. Another reference population (*n* = 496) for resistance to *V. alginolyticus* was established in this experiment, which derived from the unselected breeding population that had no genetic connection to GS3 line. The selection index was calculated based on the genomic estimated breeding value (GEBV) of the two disease traits, where each GEBV was standardized and given a weighting of 0.5. Candidates with the superior comprehensive index ranking were selected from the GS3 line in F2 generation stocks population (*n* = 560), resulting in the creation of the disease-resistant breeding line titled GS9. In addition, the GS3 breeding line underwent continued genomic selection for resistance to *C. irritans* and produced the F3 progeny. Simultaneously, two populations (C1 and C2) that had not undergone selective breeding were used to produce offspring as the control group. Finally, the genetic improvement effect of all breeding lines (GS3, GS9, C1, C2) was assessed through artificially challenge experiments with *C. irritans* and *V. alginolyticus* by using their offpsring, respectively. For further details on the application of genomic selective breeding program, please refer to Figure 1.

### 2.3 Built up the standard artificial challenge for *Vibrio alginolyticus*

Before to conducted the genomic selection breeding of resistance to double traits by using the selection index, three preliminary experiments on large yellow croaker resistance to *V. alginolyticus* were carried out to determine the artificial infection method, *V. alginolyticus* strain and concentration, respectively. *V. alginolyticus* was provided by Fujian Academy of Agricultural Sciences (Deposit No.: H2017041302).

The first experiment was to explore the effects of immersion infection and intraperitoneal injection on resistance to *V. alginolyticus* in large yellow croaker. For the immersion experiment, 406 fish (body weight (g): 5.2 ± 1.7, body length (cm): 7.10 ± 0.82) were randomly sampled from the marine aquaculture cages and transported to the 8 tons cement pond. After a week of acclimation, fish were divided into three groups and placed into respective tanks, where a seawater level of 200 L maintained during the infection, and infected by immersion for 2 h in a bacterial suspension (1.0 × 10^5^ CFU/mL). The immersion infection experiment lasted for 6 days, and the observation and recordings were made every 2 h. For the intraperitoneal injection experiment, 397 fish (body weight (g): 6.26 ± 1.84, body length (cm): 7.13 ± 0.79) were divided into three groups, anesthetized with tricaine methanesulfonate at a concentration of 80 ppm (MS222; Sigma, St. Louis, MO, USA), and artificially injected with 0.1 mL of *V. alginolyticus* MTH strain at a concentration of 1.0 × 10^9^ CFU/mL. In additional, 30 fish from control group were injected with 0.1 mL of normal saline solution. The experiment was continuously observed, and the mortality was recorded for 48 h. Survival time and survival status were used as indicators of the antibacterial resistance of large yellow croaker during artificial infection challenges. For the survival status trait, surviving fish were recorded as 1, while dead fish were recorded as 0. If a fish loses its balance after infection and does not recover within 10 s, the corresponding time is recorded as the survival time, and the survival status is indicated as 0 If a fish remains alive and in good condition 48 h after infection, it is considered to have strong disease resistance, and the survival status is recorded as 1. This standard was adopted in subsequent experiments to assess the disease resistance of large yellow croaker.

The second experiment was to identify the strains suitable for large yellow croaker to infect *V. alginolyticus* artificially. Four strain named CZH, DWH, MTH and SXJH were randomly selected for this experiment. The concentrations of the four strains ranged from 5.3 to 6.2 × 10^9^ CFU/mL, and each strain infected 37 to 42 fish (body weight (g): 9.68 ± 2.86), with an injection dose of 0.1 mL for each fish. The mortality of experiment was continuously observed and recorded for 48 h.

After the infection strain of *V. alginolyticus* was confirmed, the third experiment was conducted to ascertain the appropriate concentration level of *V. alginolyticus* infection in large yellow croaker. The initial strain concentration (6.78 × 10^9^ CFU/mL) was diluted 2, 5, 25 and 125 times using normal saline solution (corresponding to 3.4 × 10^9^, 1.3 × 10^9^, 2.7 × 10^8^, 5.4 × 10^7^ CFU/mL, respectively). An average of 22 fish were infected with each diluted concentration (using 0.1 mL injection), and the experiment was continuously observed for 48 h.

### 2.4 Construction of reference population

After determining the *V. alginolyticus* strain, challenge method and concentration, 708 fish (body weight (g): 10.75 ± 3.70, body length (cm): 9.02 ± 1.05) were randomly selected from an unselected breeding population and divided into four cylindrical tanks (1 m^3^). The fish were anesthetized with MS222 and intraperitoneally injected with MTH *V. alginolyticus* strain (3.16 × 10^8^, 0.1 mL). The experimental water temperature was maintained at 30°C, and mortality was continuously observed and recorded for 48 h that used as the reference population phenotype data of resistance against *V. alginolyticus*. The fish lost their balance and could not recover within 10 s, which meant that the fish had a weak resistance to *V. alginolyticus*. On the contrary, 48 h after infection, the fish were still alive and showed good vitality, which indicated that the fish had strong antibacterial properties. Therefore, both survival time and survival state were used as the indicators of resistance performance. Samples (*n* = 496) were randomly selected from the phenotype reference population for genotyped.

### 2.5 Genotyping and quality control

Samples (*n* = 496) were randomly selected from the resistance against *V. alginolyticus* reference population and 560 (body weight (g): 664.20 ± 159.47, body length (cm): 32.94 ± 3.04) healthy broodstock fish were randomly selected from F2 generation (GS3) strain which was undergoing two rounds of selective breeding for resistance to *C. irritans*. Meanwhile, 415 broodstock fish (body weight (g): 812.20 ± 122.20, body length (cm):32.94 ± 3.04) were randomly selected from the unselected breeding population as the control group (C2). Fin samples of these reference and broodstock fish were collected and genotyped by using “NingXin-III” 55 K genotype array (17) in MolBreeding Biotechnology Co., LTD (Shijiazhuang, HeBei). The raw genotype data of reference and candidate population were obtained and the quality control of the genotypes was conducted using Plink (version 1.9) software (18) with the criteria of individual call rate > 0.9, minor allele frequency > 0.05, SNP call rate > 0.9. The quality genotype data was used for next analysis. The CMplot (version 4.5.1) package (19) of R software was used to map the probability density distribution of SNP loci in the genome. The genome referenced for this analysis is available at https://www.ncbi.nlm.nih.gov/datasets/genome/GCA_003845795.1/.

### 2.6 Genetics analysis of resistance to *Vibrio alginolyticus*

The univariate mixed linear model of GEMMA 0.94.1 software (20) was used to conduct genome-wide association analysis (GWAS) on the survival time and survival status traits of large yellow croaker resistant to *V. alginolyticus*, respectively. The tank effect was added to the model as a fixed effect in the univariate mixed linear model. The *p*-value threshold of 0.0001 was taken as the significance association threshold. The 50 kb upstream and downstream regions of significant SNPs were identified as candidate regions, and R software was used to screen related genes in these candidate regions. The results of genome-wide association analysis were plotted by qqman package (21) of R software. The heritability of survival time trait of large yellow croaker for resistance to *V. alginolyticus* was calculated by using HIBLUP software (22). In additional, the genetic correlation between resistance to *V. alginolyticus* and *C. irritans* trait in large yellow croaker was also calculated based on the GEBV of two reference population.

### 2.7 Construction the resistance strain and control group

The reference population (*n* = 474) with the phenotype of resistance to *C. irritans* in large yellow croaker was constructed in the laboratory earlier, and genotype by 55 K SNP array. Another reference population (*n* = 496) that resistance to *V. alginolyticus* was built up in this experiment. Based on the two references genotype and phenotype data, the GEBV of two candidate populations of large yellow croaker (GS3-F2 broodstock (*n* = 560) and unselected broodstock fish named C2 (*n* = 415)) was calculated by using the BayesB model in the BGLR package in R software (23). The final selection index was calculated based on the standardized GEBV of each disease trait and with a weighting of 0.5, respectively.

Based on the GEBV ranking of resistance to *C. irritans* in GS3 breeding line F2 broodstock fish, 50 individuals with the top ranking were selected as the candidate parents for produced the F3 offspring (GS3-F3). Additionally, the top 50 ranking individuals were selected from the GS3 breeding line F2 broodstock fish based on the selection index and produced the F1 generation of double trait breeding line (GS9-F1). If the screened GS9 candidate was the same as the GS3 candidate, the GS3 candidate was given priority. Control groups were derived from two different unselected breeding candidate populations, with one being genotyped for genetic improvement of swimming performance traits. The GEBV of the selected candidate parents for two disease resistance traits was also calculated. All candidate group were being randomly mating to produce offspring in January 2022. According to the culture standard of large yellow croaker (NY/T 5061–2002), the fish in each group were fed for 2 months in a cement pond with 100 tons of water, with the water temperature kept at 24°C, oxygen concentration maintained at 5.0 mg/L, and water changed daily to remove waste.

### 2.8 Offspring challenge test

The fish were randomly selected from each group for the *C. irritans* infection experiment. The number of challenged fish in each group was as follows: GS9 (body weight (g): 2.97 ± 1.05, body length (cm): 5.32 ± 0.66), GS3 (body weight (g): 4.28 ± 1.28, body length (cm): 6.15 ± 0.62), C1 (body weight (g): 2.55 ± 0.79, body length (cm): 5.21 ± 0.63) and C2 (body weight (g):2.97 ± 1.02, body length (cm):5.39 ± 0.76), with 487, 508, 510, and 483 fish, respectively. The fish were then transported to the pond and acclimated for a week before the experiment. During this period, the individuals were fed normally and feeding was stopped 3 days before the start of the experiment. After stabilization, a challenge test for resistance to *C. irritans* was conducted in 1-ton tanks, with each group having 3 replicates. Based on the results of the preliminary challenge test, the infection concentration of *C. irritans* was set at 500 larvae per liter of water in the formal test, and the experimental water temperature was maintained at 25–26°C. Observations were recorded every 2 h, and the experiment lasted for 5 days.

In the offspring resistance to *V. alginolyticus* challenge experiment, a total of 1,566 7-month-old offspring were randomly selected from the double trait bred strain (GS9, body weight (g): 22.67 ± 6.54, body length (cm): 11.38 ± 1.21), single trait bred strain (GS3, body weight (g): 16.03 ± 3.90, body length (cm): 10.21 ± 0.84), C1 control group (body weight (g): 16.70 ± 5.55, body length (cm): 10.22 ± 1.30), and C2 control group (body weight (g): 13.82 ± 4.37, body length (cm): 9.71 ± 1.09), and then transported to the pond and acclimated for a week before the experiment. After stabilization, a challenge test for resistance to *V. alginolyticus* was conducted in 1-ton tanks, with each group having 3 replicates. Based on the results of the preliminary challenge test and the size of the fish in this experiment, 0.2 mL bacterial solution with a concentration of 3.32 × 10^7^ CFU/mL was injected into the abdominal cavity of large yellow croaker for the main experiment. The experimental water temperature was maintained at 28–29°C and data were continuously observed and recorded every 2 h for 2 days.

## 3 Results

### 3.1 Standard for artificial challenge of *Vibrio alginolyticus* infection in large yellow croaker

**Figure 2:**
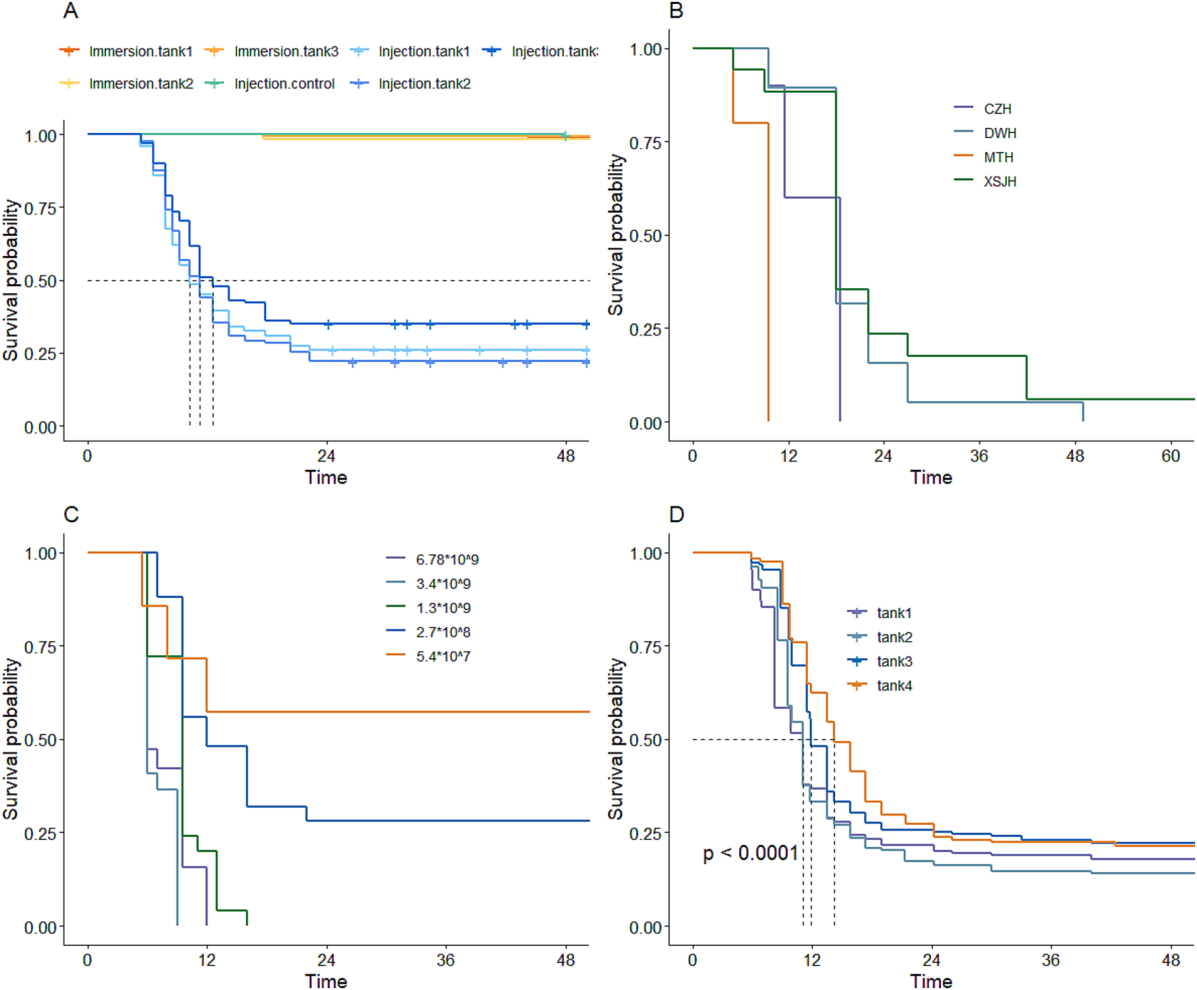
The standard of artificial infection of resistance against *V. alginolyticus* in large yellow croaker. **(A)** Challenge method. **(B)** Different strains. **(C)** Different concentrations. **(D)** Reference population constructed.

In the first preliminary experiment to determine the artificial challenge method for infecting large yellow croaker with *V. alginolyticus*, the results showed that the mortality rate was less than 2% within 48 h following the immersion infection challenge. In contrast, the fish subjected to intraperitoneal injection of *V. alginolyticus* showed a peak death rate at 6 h post-challenge, which continued until 24 h and then stabilized. Meanwhile, the control group injected with normal saline solution had no deaths within 48 h. These results indicated that intraperitoneal injection was the most effective method for challenging large yellow croaker with *V. alginolyticus* to test resistance (Figure 2A).

The results of the experiment using involving diverse strains and the intraperitoneal infection method on large yellow croaker demonstrated that the virulence of various *V. alginolyticus* strains varied. Among these strains, the MTH strain exhibited the strongest virulence, whereas the XSJH strain exhibited the weakest (Figure 2B). The experiment investigated the impact of different concentrations of MTH strain on large yellow croaker mortality rates. The results showed that the higher infection concentrations led to more rapid and substantial mortality rates, with the optimal concentration of MTH strains for the infection challenge in large yellow croaker identified as 10^7^–10^8^ CFU/mL (Figure 2C).

### 3.2 Reference and candidate population resource

To construct a phenotypic reference population of large yellow croaker resistant to *V. alginolyticus*, 708 individuals were artificially intraperitoneally injected with *V. alginolyticu* strain (MTH strain, 0.1 mL) at a concentration of 3.16 × 10^8^ CFU/mL, based on the pre-experimental results (Figure 2D). Among them, 496 individuals were randomly selected and genotyped by using 55 K SNP array, and 45,133 SNPs were obtained after quality control. The average distance between SNPs was 12.4 kb (Supplementary Figure S1). Additionally, 44,449 SNPs were obtained from the 930 candidate genotypes (523 GS3 F2 candidate parents and 407 unbred control candidate parents) after quality control.

### 3.3 Genome wide association of resistance to *Vibrio alginolyticus*

**Figure 3:**
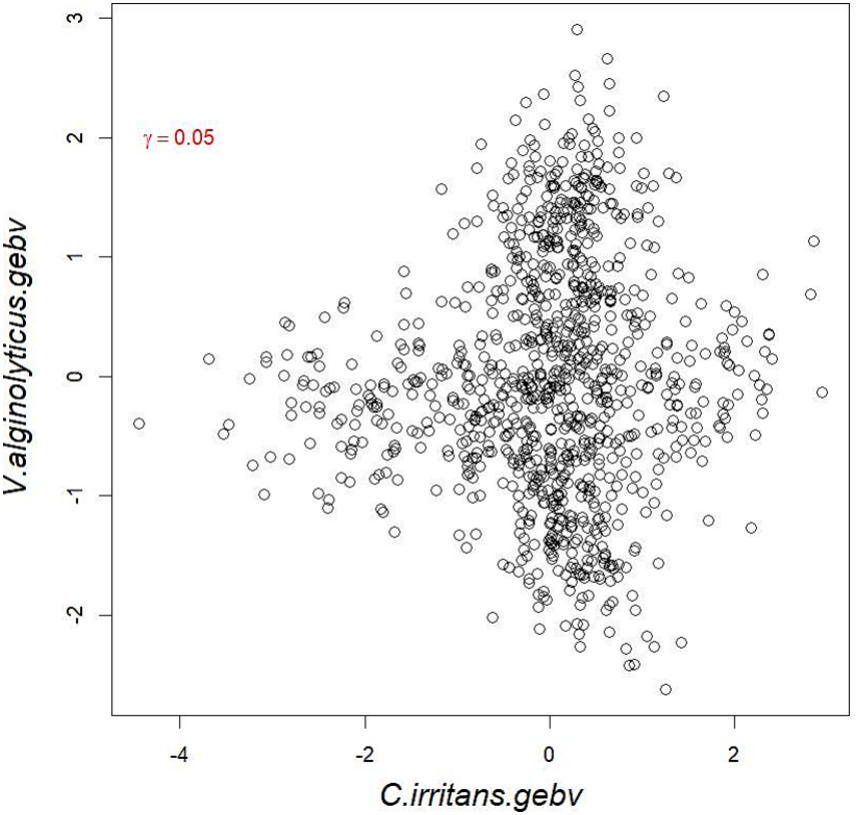
Genome-wide association analysis of *V. alginolyticus* resistance traits in large yellow croaker.

**Figure 4:**
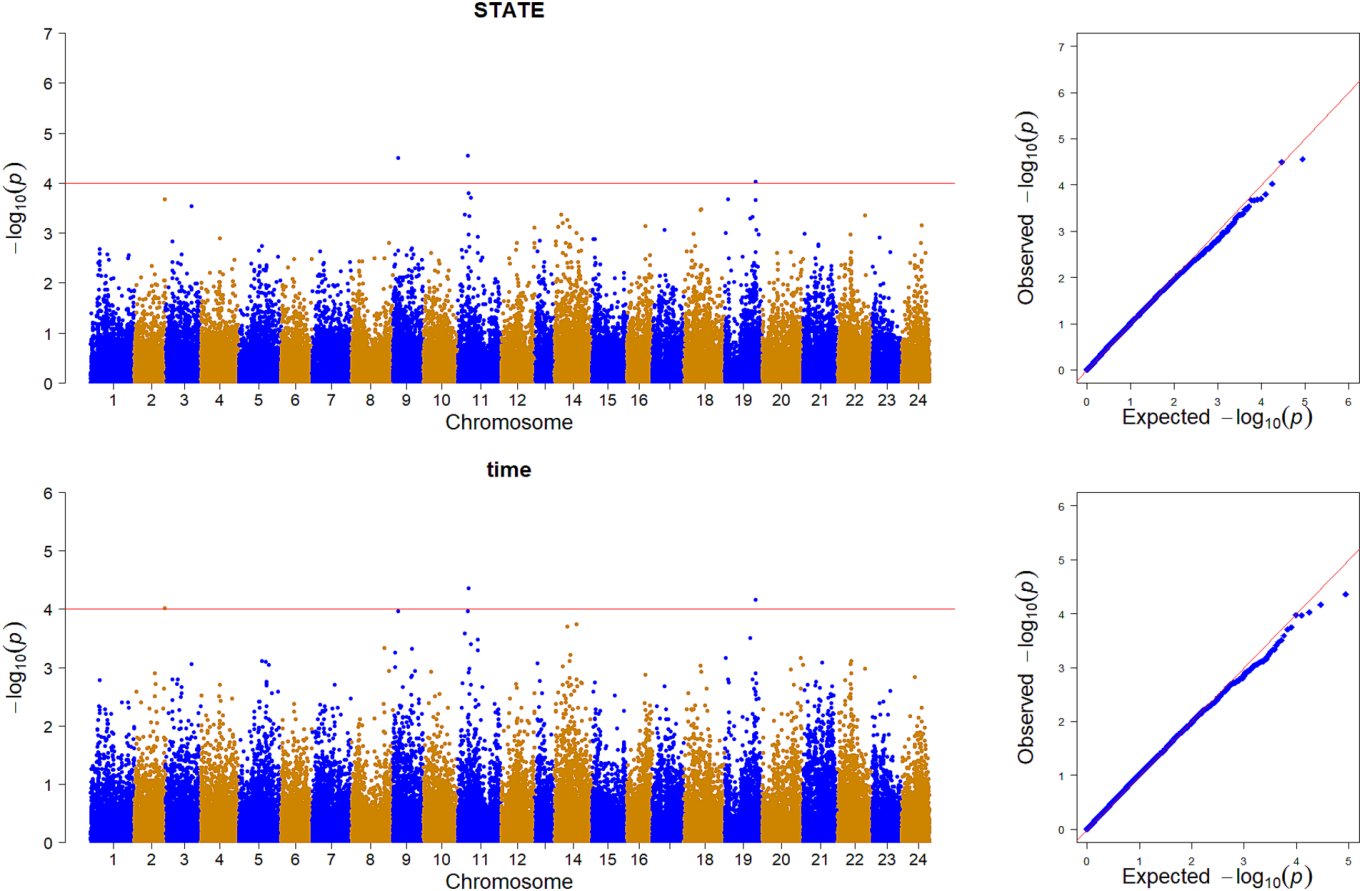
Genetic correlation between resistance to *V. alginolyticus* and *C. irritans* in large yellow croaker.

GWAS was performed by using the reference population (sample = 496) for the survival time and survival status of resistance to *V. alginolyticus* in large yellow croaker. The results showed that significant SNPs were identified in chromosome 2, 9, 11 and 19 (Figure 3). Except for the SNPs in chromosome 2, the rest of SNPs were located in the Prmt3, Fmnl2, Coro7, and Nell1 genes, respectively. The heritability of resistance to *V. alginolyticus* was approximately 0.1 (Standard error 0.008), and the genetic correlation between resistance to *V. alginolyticus* and resistance to *C. irritans* was 0.05 (Figure 4).

### 3.4 Offspring produced

**Figure 5:**
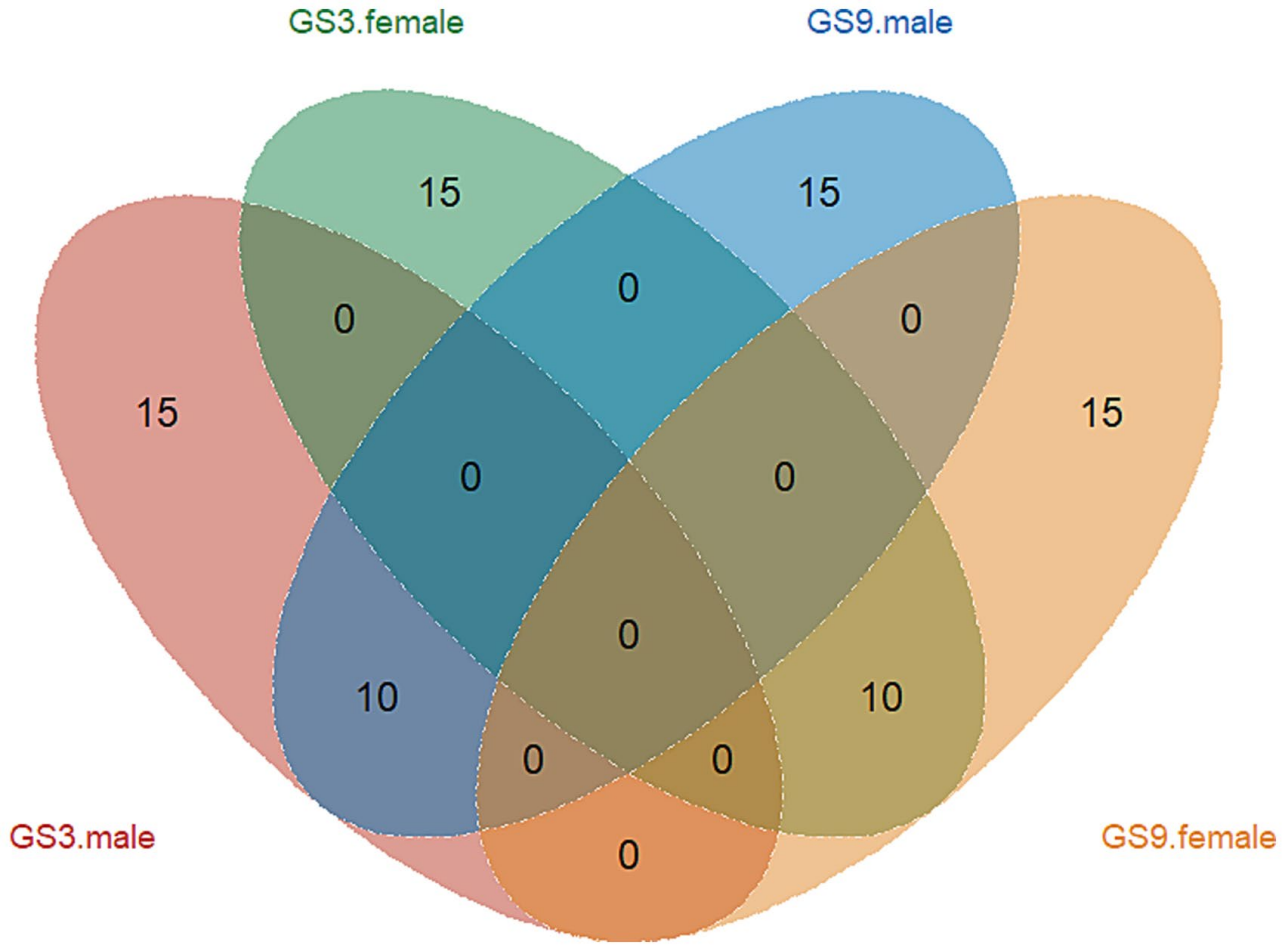
Venn diagram of GS3 and GS9 breeding line of candidates in large yellow croaker.

**Figure 6:**
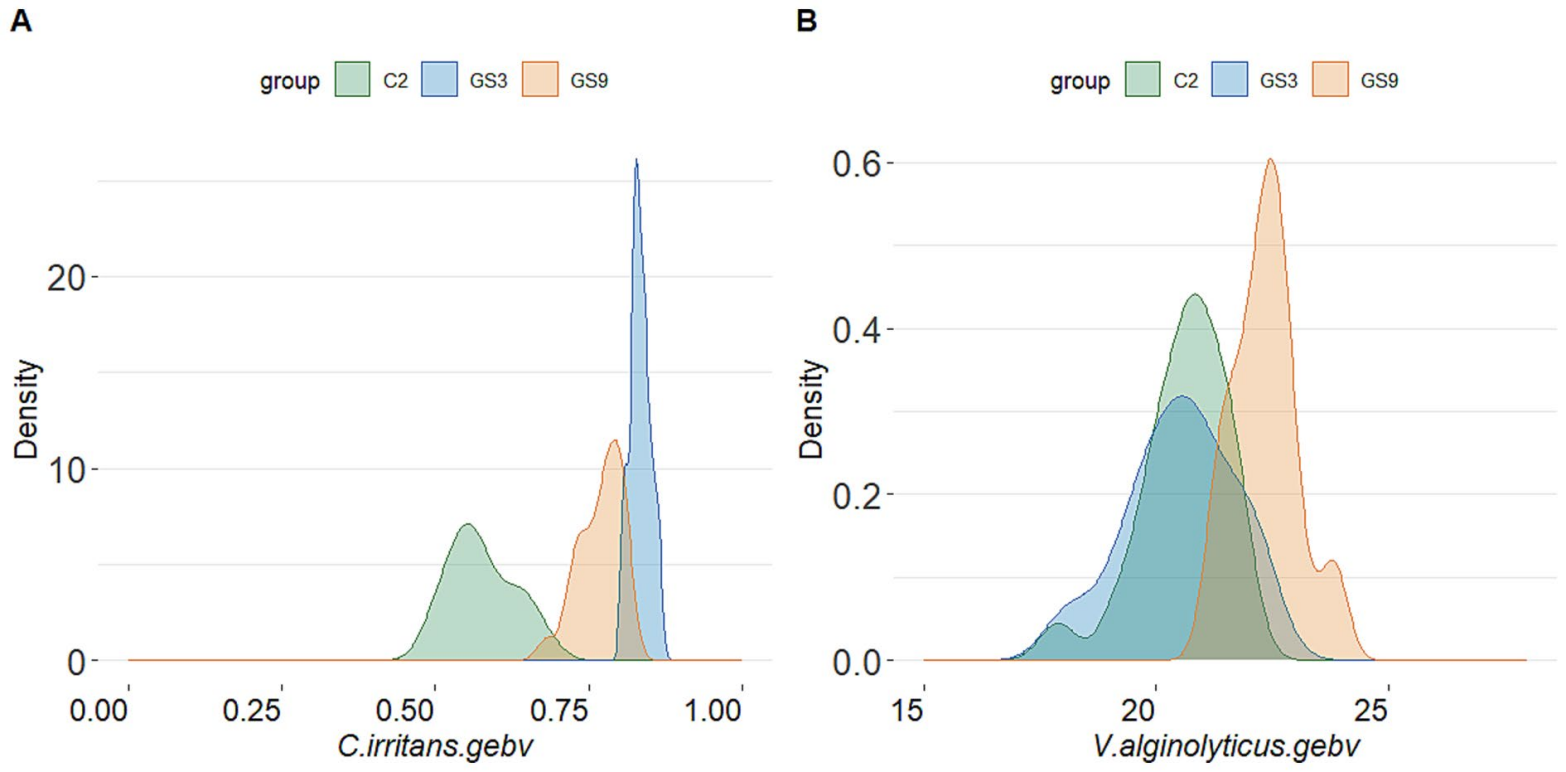
GEBV probability density of candidate fish of each breeding line of large yellow croaker. **(A)** *C. irritans*. **(B)** *V. alginolyticus*.

The GEBV and selection index were calculated by using the reference population of resistance to *V. alginolyticus* (sample = 496) and *C. irritans* (sample = 474) in large yellow croaker. Finally, 50 broodstock candidates (33 females, 17 males) with top GEBV rankings and 50 broodstock candidates (33 females, 17 male) with top selection index were selected from the GS3-F2 population. To evaluate the coincidence between the selection index and the GEBV, the top 25 male and female candidates ranked in GEBV and selection index were selected for further analysis. The results showed that 10 individuals of each sexoverlapped (Figure 5). The average GEBV of resistance to *C. irritans* traits of GS3, GS9 and C2 candidates were 0.83, 0.77 and 0.58, respectively. For resistance to *V. alginolyticus* trait, the average GEBV of GS3, GS9 and C2 broodstock were 20.53, 22.35 and 20.60, respectively (Figure 6). The GS3, GS9, and C2 broodstock produced 2.7 kg, 3.1 kg, and 4.0 kg of floating eggs, respectively. After 2 months of standard culture feeding in a 100 m^3^ pond, approximately 500 thousand, 576 thousand and 480 thousand juvenile fish of the GS3, GS9, and C2 strains, were released into marine cages, respectively (Table 1).

**Table 1:** GWAS result of resistance against *V. alginolyticus* in large yellow croaker.

Trait	Chr	Position	*p*-value	Distance (±kb)	Description	Gene
State	11	8,393,559	4.558	Gene	Protein arginine N-methyltransferase 3	PRMT3
	19	24,644,691	4.027	Gene	Formin-like protein 2”	Fmnl2
	9	4,275,586	4.497	Gene	Coronin-7”	CORO7
Time	11	8,784,958	4.357	Gene	Protein kinase C-binding protein NELL1”	NELL1
	19	24,644,691	4.162	Gene	Formin-like protein 2”	Fmnl2
	2	23,944,376	4.017	3.271	—	—

### 3.5 Offspring challenge test

**Figure 7:**
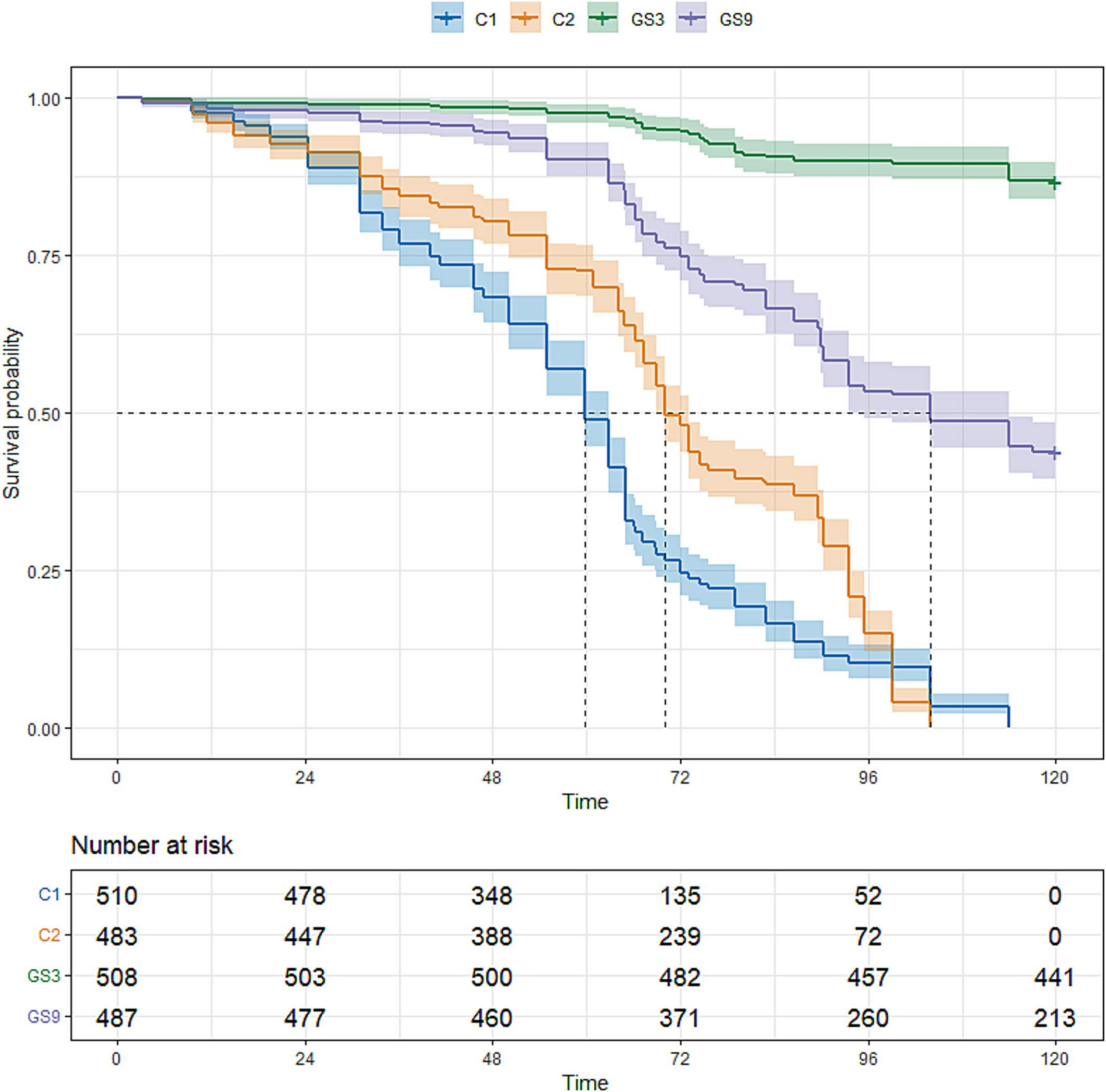
Result of resistance to *C. irritans* of large yellow croaker breeding lines.

**Figure 8:**
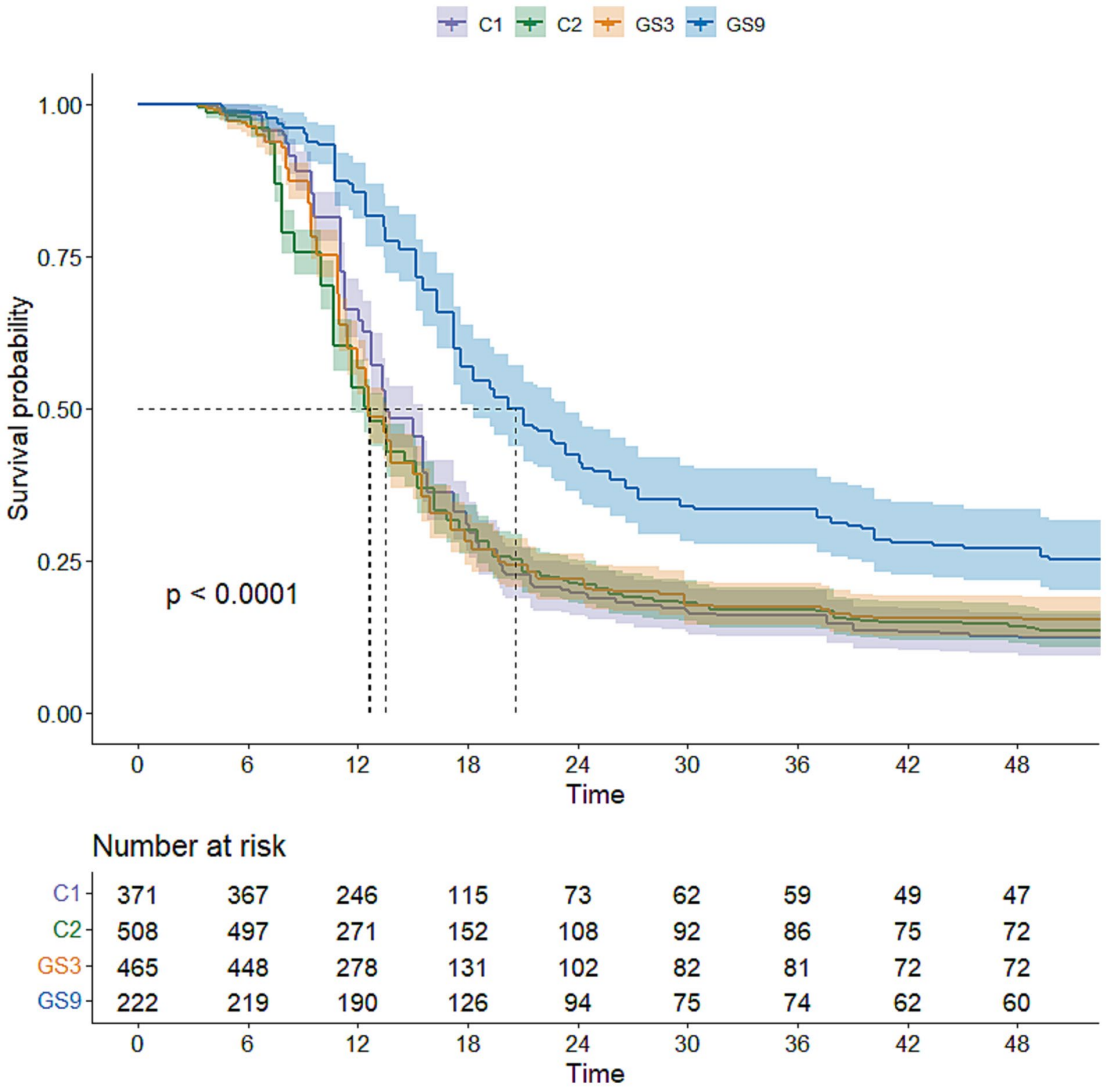
Result of resistance to *V. alginolyticus* of large yellow croaker breeding lines.

To evaluate the genetic improvement of resistance to *C. irritans* in the double-trait bred strain and single-trait bred strain, 487, 503, 510 and 483 5-month-old fish selected from GS9, GS3, C1 and C2, respectively, were tested using an artificial infection challenge. The survival rates of GS9 and GS3 were 43.74 and 86.81%, respectively, after 120 h of challenge, whereas those of C1 and C2 remained at 0 % (Figure 7). Additionally, 222, 465, 371 and 508 8-month-old fish from GS9, GS3, C1 and C2 were tested in a *V. alginolyticus* infection experiment. The survival rates of GS9 and GS3 were 27.03 and 15.48%, respectively, while the survival rates of C1 and C2 were 12.67 and 14.17% (Figure 8).

## 4 Discussion

### 4.1 Standard for artificial challenge of *Vibrio alginolyticu* infection in large yellow croaker

The objective of this study was to explore the effect of genetic improvement of double traits in resistance to *V. alginolyticus* and *C. irritans.* Before implementing genomic selection for both disease traits, it is imperative to establish standardized artificial challenges of *V. alginolyticus* resistance in large yellow croaker and build up a reference population. In studies on artificial challenge infection of aquatic species with bacterial diseases globally, the majority have used bacterial solution injection as the method for infection challenge. For instance, Yan et al. injected *V. alginolyticus* into the abdominal cavity of large yellow croakers (body weight: 300–500 g) to investigate its immune efficacy. They found the number of red blood cells, white blood cells, and lymphocytes in the infected group was significantly higher compared with the control group, while the number of granulocytes in peripheral blood was significantly lower than control group 1 day after the injection. Antibody titer began to increase on day 7 and reached its peak on day 15 (24). Fevolden et al. evaluated the resistance performance of individuals with high and low stress levels by injecting vibrio into rainbow trout (*Oncorhynchus mykiss*) (25). Leeds et al. injected *Flavobacterium psychrophilum* into rainbow trout to explore the improvement of rainbow trout resistance to bacterial cold water disease (BCWD) (26). The mortalities record data at day 21 showed that the survival rate of select-line increased by 24.6% after one generation of selection and by 44.7% after two generations of selection. Fraslin et al. found that immersion and injection affected the genetic mechanism of resistance to *Flavobacterium psychrophilum* in rainbow trout, which might be caused by epistatic interactions between resistance QTL (27). The result showed that the overall survival rate was 77% on day 48 for immersion infection and 55% survival rate on day 34 for injection challenge. Additionally, regardless of the route of infection, fish body weight at the time of infection did not have a significant effect on either time to death or survival rate. Degremont et al. injected *Vibrio aestuarianus* into Pacific oysters (*Crassostrea gigas*) offspring to evaluate the resistance performance of selectively bred strains (28). The effect of two infection methods (immersion and intraperitoneal injection) was compared in large yellow croaker in this study, and results showed a significant difference in challenge test between two infection methods, revealing that intraperitoneal injection is more suitable for resistance to *V. alginolyticus* in large yellow croaker. Unlike the *C. irritans* infection expreiment in large yellow croaker, intraperitoneal injection of *V. alginolyticus* is an acute infection experiment with short experiment period and fast death rate in our study, which is consistent with the research showing that non-specific immune factors in large yellow croakers played an important role on the first day after injection of *V. alginolyticus* (24). Furthermore, a study showed that intraperitoneal injection of *V. alginolyticus* in Pacific white shrimp (*Litopenaeus vannamei*) significantly increased its non-specific immune antibacterial proteins within 12 h (29, 30). The multiple injections experimental results in large yellow croaker showed that the injection concentration could significantly affect the survival rate of large yellow croaker infected with *V. alginolyticus*. When the injection concentration was 10^9^ CFU/mL, all fish died within 12 h. At an injection concentration of 10^8^ CFU/mL, the 24-h survival rate of experimental fish was 25% when the injection concentration was 10^7^ CFU/mL, the 24-h survival rate of experimental fish exceeded 50%. Therefore, for the specifications of large yellow croakers in this experiment, the optimal concentration of MTH strains for the infection challenge in large yellow croaker identified as 10^7^-c10^8^CFU/mL. Consequently, prior to formal *V. alginolyticus* infection experiments, preliminary trials are necessary to determine the appropriate dose and concentration.

### 4.2 Genetic analysis of resistance to *Vibrio alginolyticus* infection in large yellow croaker

As is well known, traits with high heritability often exhibit substantial genetic variation, indicating a significant potential for trait improvement. However, most disease resistance traits in aquaculture exhibit low to medium heritability. For example, the heritability of resistance to *V. alginolyticus* ranged from 0.133 to 0.257 (31) in Pacific Oyster (*Crassostrea giga*). The heritability of resistance to *Streptococcus agalactis* of tilapia ranged from 0.23 to 0.25 (32), and the heritability of resistance to BCWD of rainbow trout ranged from 0.22 to 0.35 (26). In this study, the heritability of survival time trait related to *V. alginolyticus* resistance in large yellow croaker was 0.1, indicating it as a low heritability trait. This means that it is difficult to screen out resistant individuals through pedigree selection for genetic breeding of large yellow croaker against *V. alginolyticus*, making genomic selection technology more suitable for breeding applications targeting this disease trait (33). Moreover, this may require multiple generations of continuous genetic improvement to achieve significant genetic progress. Based on the phenotypic data and genotype data of the reference population of *V. alginolyticus* resistant established by random population, genome-wide association analysis was carried out and the results showed that resistance to *V. alginolyticus* in large yellow croaker is a polygenic trait. Only a common SNP was identified on both survival status and survival time traits. This SNP is located in the Fmnl2 gene in chromosome 19, which encodes a formin protein involved in cytoplasmic division and cell polarity. Compared with random population, the genetic structure of family population may powerful for the genome-wide association analysis to identified the candidate gene related with the important trait. For example, Sukhavachana et al. conducted genome-wide association analysis on *Streptococcus agalactis* traits based on tilapia family population data, and the SNP loci located on chromosomes 11 and 16 could explain 10% genetic variation of this trait (34). Vallejo et al. conducted genome-wide association analysis of BCWD trait in rainbow trout based on family population data, and identified 9 significant QTL regions, which could explain 88% genetic variation (35). Yang et al. identified 3 QTL association with *V. alginolyticus* resistance trait in Pacific oysters based on family population data (36).

Prior to applying the genetic improvement of multi-trait, it is essential to evaluated the genetic correlation between each trait. Positive correlations among the traits show that the changes of two traits are in the same direction, while negative correlations indicate two traits cannot be improved by breeding at the same time, and strategies like index selection or multi-trait selection models can help identify optimal compromises. However, for most aquatic species, the complex underwater environment greatly increases the difficulty of acquiring disease traits and it is impossible for the same individual to be artificially infected with both diseases at the same time, which means that the phenotypic correlation between two disease traits is difficult to accurately calculate. In this case, most studies have calculated genetic correlations based on family and half-sib populations to reflect the relationship between two disease traits. For example, the genetic correlation between BCWD and columnaris disease in rainbow trout was 0.39 ~ 0.40 based on family population data (37). Similar results were also been found in Evenhuis et al. study that the genetic correlation and phenotypic correlation was 0.35 and 0.2, respectively (38). The genetic correlation between the resistance of rainbow trout to *Piscirickettsia salmonis* and sea louse resistance (*Caligus rogercresseyi*) is 0.39, which is possible to improvement of these two disease resistance traits simultaneously (39). In Atlantic salmon, the genetic correlation between *Aeromonas salmonicida* infection resistance and anemia traits was 0.15 in a family population (40). Gjedrem et al. found the genetic correlation between the ability to survive the diseases for the furunculosis, bacterial kidney disease and BCWD in Atlantic salmon were all positive (41). In this study, considering the high cost of constructing families of large yellow croaker, the genetic correlation of two disease traits was calculated based on the genotypic and phenotypic data from two reference populations. The results showed that there was a weak positive correlation between *V. alginolyticus* resistance and *C. irritans* resistance of large yellow croaker, which supports the application of a selection index for these two disease traits.

### 4.3 Disease resistance performance of double trait strain

In this study, the double-trait strain was constructed based on the selection index in F2 generation of *C. irritans* resistance strain (GS3) in large yellow croaker. Meanwhile, the F3 generation of *C. irritans* resistance strain in large yellow croaker was also produced based on the GEBV ranking of F2 candidate population. The average GEBV of *C. irritans* resistance trait of broodstock fish was ranked as follows: GS3 (0.83) > GS9 (0.77) > C2 (0.58), indicating that the F3 offspring of GS3 strain exhibited greater resistance to *C. irritans* than those of other breeding strains. The survival rate of all breeding strain offspring in the artificial challenge infection supported this conclusion. The GS3 breeding strain had the strongest resistance to *C. irritans*, followed by GS9, while C1 and C2 exhibited weaker resistance. The survival rate of GS9 and GS3 was 43.74 and 86.81%, respectively, and that of C1 and C2 was 0% in resistance to *C. irritans*.

In terms of resistance to *V. alginolyticus*, the average GEBV of GS9 broodstock fish was the highest (22.35), while the average GEBV of GS3 broodstock fish and C2 broodstock fish were similar (20.53 and 20.60, respectively). The survival rate of GS9 offspring infected with *V. alginolyticus* was 11.55 percentage points higher than that of GS3, 14.36 and 12.86 percentage points higher than that of C1 and C2 controls, respectively. These findings was consistent with the average GEBV ranking of all candidate fish strains. All experimental groups showed good repeatability in the artificial infection challenge of two diseases (Supplementary Figures S2, S3). Similar results have been found in the genomic selection breeding of tilapia against *Streptococcus agalactiae*. The average GEBV of the candidate fish of the breeding strain is higher than that of the control group, and the survival rate of the offspring of the breeding strain is 21% higher than that the control group (32).

The performance of artificial infection experiment of the breeding strain offspring was consistent with the expectations. However, the disease risk and challenge of large yellow croaker cultured in sea area are much higher than the experimental conditions of land-based artificial infection experiment. Only the survival rate and growth performance and other indicators observed in the cultured sea area can accurately reflect the genetic improvement effect of all breeding strains. Although tracking this trait takes more time, energy and cost, it has important breeding significance and value for the genetic improvement application of large yellow croaker.

## 5 Conclusion

This study establishes the technical standard for artificial infection of *V. alginolyticus* in large yellow croaker, and selectively breeding for the double trait of resistance to *V. alginolyticus* and *C. irritans* by using the selection index based on the *C. irritans* resistance strain in large yellow croaker. The experiment on artificial infection challenge demonstrated a significant and simultaneous improvement in the survival rate of the double trait strain in resistance to both *C. irritans* and *V. alginolyticus*. In contrast, the single-trait selection strain only demonstrated a significant improvement in resistance to *C. irritans*. These results indicate that genetic improvement of the double trait in large yellow croaker using genome selection technology is a viable approach. This provides a reference for the application of double trait breeding in aquatic species.

## Data Availability

The datasets presented in this article are not readily available because the data (genotypes and phenotypes) are part of a reference population used for genomic selection and have commercial value. Requests to access the datasets should be directed to corresponding author.

## Supplementary material

SUPPLEMENTARY FIGURE S1 SNP density distribution of large yellow croaker reference population.

SUPPLEMENTARY FIGURE S2 Result of resistance to *C. irritans* in each replicate of large yellow croaker.

SUPPLEMENTARY FIGURE S3 Result of resistance to *V. alginolyticus* in each replicate of large yellow croaker.

SUPPLEMENTARY TABLE S1 The situation of offspring of different breeding line of large yellow croaker.
