# Supplementary figures and images for: Application of selection Index for enhancing resistance to Cryptocarya irritans and Vibrio alginolyticus in large yellow croaker
Source: Front Vet Sci. 2025 Jan 17;12:1524914. doi: 10.3389/fvets.2025.1524914 (PMC11782216; doi:10.3389/fvets.2025.1524914)

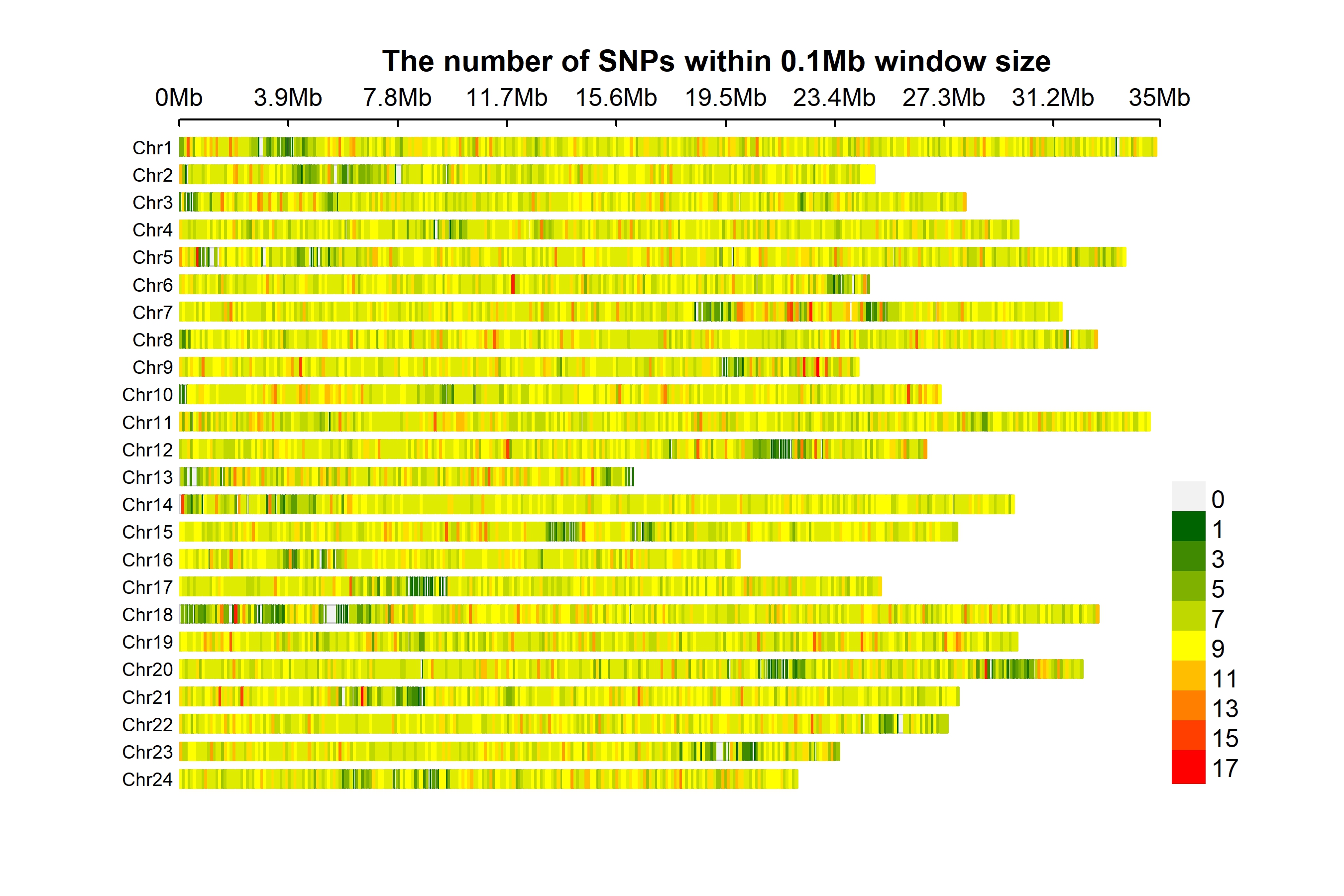

Supplement: SUPPLEMENTARY FIGURE S1 — SNP density distribution of large yellow croaker reference population. [file Image_1.JPEG]

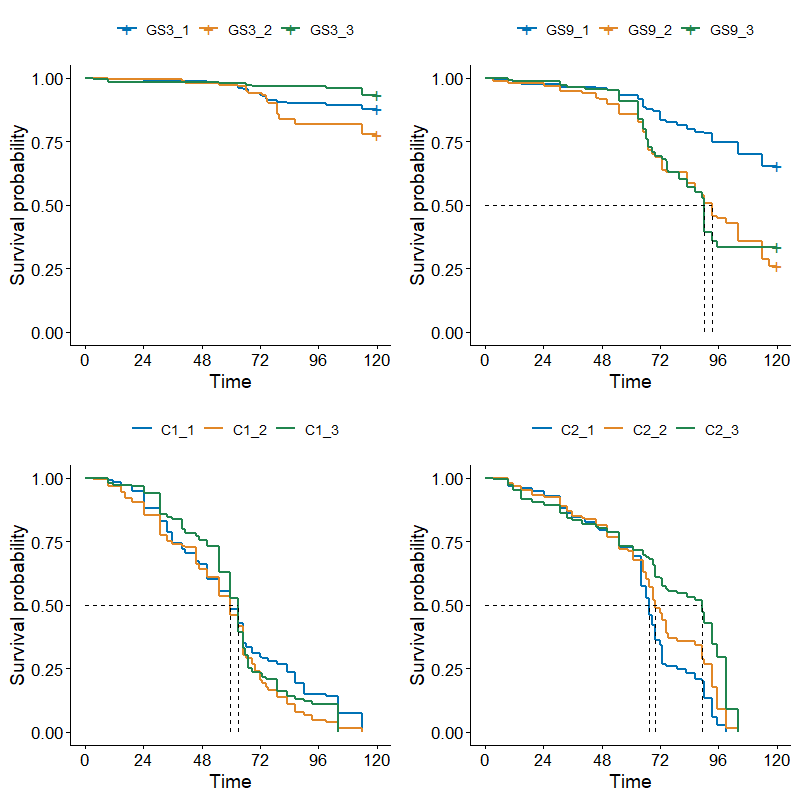

Supplement: SUPPLEMENTARY FIGURE S2 — Result of resistance to C. irritans in each replicate of large yellow croaker. [file Image_2.TIFF]

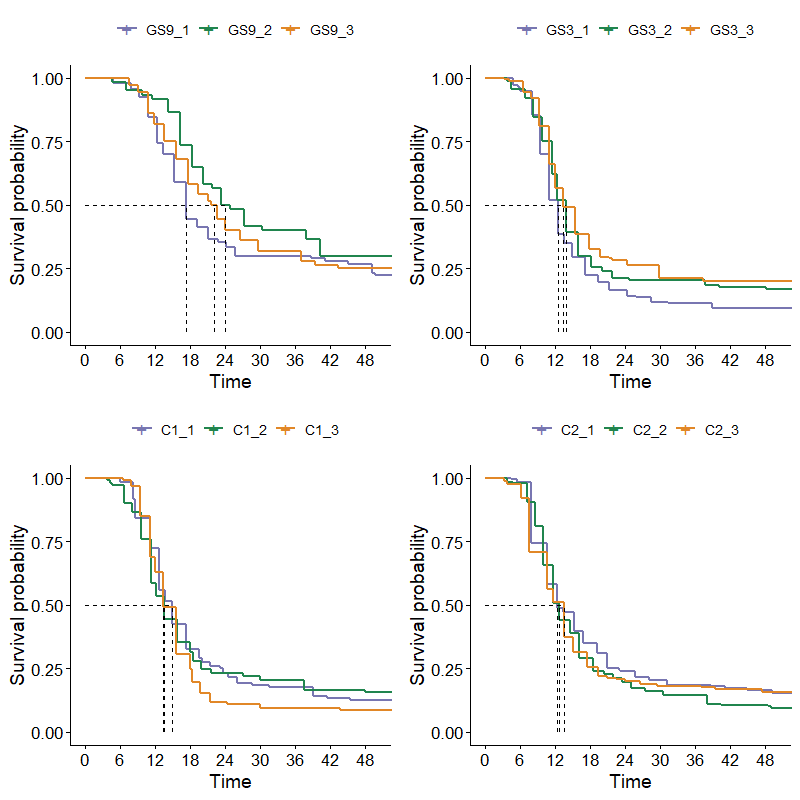

Supplement: SUPPLEMENTARY FIGURE S3 — Result of resistance to V. alginolyticus in each replicate of large yellow croaker. [file Image_3.TIFF]
